# Blood glucose response to a calamansi drink in healthy adults: a non-randomised study

**DOI:** 10.1186/s13104-020-05250-8

**Published:** 2020-08-28

**Authors:** Angela Siner, Mauna Sree Sevanesan, Tati Ambomai, Zakiah Abd. Wahab, Liwan Lasem

**Affiliations:** Department of Basic Medical Sciences, Faculty of Medicine and Health Sciences, Universiti Malaysia Sarawak, 94300 Kota Samarahan, Sarawak Malaysia

**Keywords:** Calamansi, Glycaemic index, Post-prandial blood glucose

## Abstract

**Objective:**

Glycaemic Index (GI) ranks the body’s response to carbohydrate content in food such that high GI food increases postprandial blood glucose levels. One of the popular drinks at food and beverage outlets is a drink made from calamansi, a citrus that is believed not to induce an increase in blood glucose levels. In this non-randomised single-blind (participants) study, capillary blood from 10 healthy males were sampled following consumption of either glucose or the calamansi drink. The blood glucose measurements were then used to calculate the GI for the drink.

**Results:**

The GI of the calamansi drink tested was calculated as 37, a value within the range of low GI foods.

*Trial registration* Clinical Trials identifier NCT04462016; Retrospectively registered on July 1, 2020.

## Introduction

*Diabetes mellitus* is becoming a major public health concern worldwide [1]. Prolonged hyperglycaemia increases the risk of microvascular damage such as neuropathy that contributes to increased macrovascular complications such as ischaemic heart disease and ultimately reduced life expectancy [2]. As diabetics have increased hunger and food intake partly due to accelerated gastric emptying caused by absent or delayed secretion of insulin [3], normalising blood glucose slows its progression and prevents the development of complications [4]. Low glycaemic index (GI) food with a GI of 55 or lower, are slowly absorbed and produces lower peaks in blood glucose, which is useful for maintaining glycaemic control [5]. Decreased rate of glucose absorption reduces post-prandial rise in gut hormones such as incretins and insulin by maintaining suppression on free fatty acids (FFA) and counter regulatory responses, while at the same time achieving lower blood glucose concentrations. Over time, glucose is withdrawn from the circulation at a faster rate such that its levels return to baseline despite continued absorption from the gut [6].

Calamansi, which is also known as “calamondin” in America or “limau kasturi” in Malaysia [7], is consumed by many due to its potential health benefits [8] that includes the potential to lower post-prandial blood glucose [9]. Blood glucose response following consumption of a commercially sold calamansi drink was evaluated in this study. Data from this study will be helpful to consumers making drink choices in view of the increasing number diabetic individuals in the community [10]. Data from this study will also provide baseline information for further community-based investigations related to the GI of other foods.

## Main text

### Materials and methods

This non-randomised, single-blind (participants) study that was based on the report of a joint consultation between the Food and Agriculture Organization (FAO) and World Health Organisation (WHO) [11] was conducted between January and May of 2018 at the teaching laboratory of the Faculty of Medicine and Health Sciences, Universiti Malaysia Sarawak. Consenting 18 to 19-year-old males (n = 16) were pre-screened. Although all met the inclusion criteria, 10 were selected by simple randomisation for this two-arm study. All 10 participants were advised to abstain from alcohol and sleep for at least 6–8 h; fast for 8–10 h prior to each test. During each test, participants refrained from any vigorous activity that can alter blood glucose values; drinking water was provided throughout all tests. A total of 4 tests were conducted [11]: three for glucose (i.e., reference drink) and one for calamansi drink (i.e., test drink); tests were conducted within 1–2 weeks after the previous test. Each test lasted for 2 h. The calamansi drink used in this study was obtained from a commercial source (produced and marketed mainly in Sarawak, Malaysia). During the tests, participants were given unmarked drinking containers, in which both drinks had similar odour and appearance. The same 10 participants took part in all 4 tests.

Weight was measured using a digital weighing scale (Guardian Classic Digital Weighing Scale, Malaysia); height measured using a stadiometer (SECA, 213 Hamburg, Germany). The body mass index (BMI) was calculated using the weight (in kilograms) divided by height (in meter squared) formula and interpreted using the standard weight status categories for adults 18 years old and older [12]. Waist circumference [13]; urine dipstick test using Combur-Test^®^ strips (Roche Diagnostics GmbH, Germany); capillary blood glucose levels measured using Accu-Chek^®^ glucometer (Roche, Germany) with finger prick blood obtained using Accu-Chek^®^ lancets (Roche, Germany) were also recorded [14].

Post-prandial blood glucose response measurements were done based on FAO/WHO [11]. Briefly, urine and capillary blood was measured at time “0”. The second measurement was taken 30 min post consumption of the reference drink, 75 g of glucose (Glucolin^®^, Malaysia) dissolved in 250 mL of drinking water. Urine and capillary blood were again tested at every 30-min intervals until the 120th min. The same procedures were repeated with the test drink, 250 mL of a commercially sourced calamansi drink.

The incremental area under the curve (IAUC) was used to calculate the area under the curve by applying the trapezoid rule in which the IAUC for the reference drink (glucose) was divided by the IAUC for calamansi followed by multiplication with 100 [11]. When a blood glucose value falls below the baseline, only the area above the fasting level is included and the area of the curve that was beneath the fasting concentration was excluded in the calculation. The following formula was used to calculate the GI [11]: 100 x (IAUC for test drink/IAUC for glucose drink).

### Results and discussion

All 10 participants were 19-year-old males and non-smokers with mean weight of 64.5 kg ± 8.8 (standard error, SE); mean height of 169.02 cm ± 5.2; mean BMI of 22.50 kg/m^2^ ± 2.0; mean waist circumference of 77.7 cm ± 7.8; mean random blood glucose of 4.89 mmol/L ± 0.4; negative urine dipstick result. The pre-screen ensured the participants were suitable to take part in the study as their BMI was within the ranges of 18.5 to 24.99 kg/m^2^ [12]; waist circumference was not more than 102 cm [13] and random blood glucose were less than 7.8 mmol/L [2]. None of them were on any medications; none had family history of inherited diseases and none were diagnosed with pre-existing conditions such as human immunodeficiency virus (HIV) infection, hepatitis, inflammatory bowel diseases, diabetes mellitus, heart conditions (angina, arrhythmia or heart failure), kidney disease, blood disorders such as thalassaemia [6]; none had a history of acute medical or surgical event within the past 6 months.

**Fig. 1:**
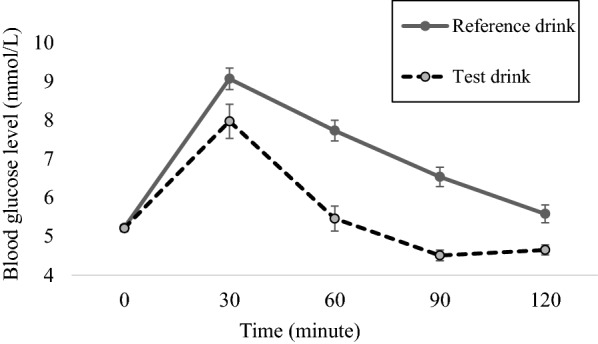
Post-prandial blood glucose levels following consumption of the reference drink (glucose) or test drink (calamansi). Each point is the average blood glucose from 30 measurements for the reference drink and the average of 10 measurements for the test drink

As shown in Fig. 1, post-prandial blood glucose at 30 min after consumption of either reference drink (glucose) or test drink (calamansi) showed that blood glucose peaked at 9 mmol/L for reference drink (glucose) and 7.9 mmol/L for test drink (calamansi). This response was similar to studies with coconut water, custard apple, cashew and soursop [15]. At 1 h post-prandial, blood glucose levels started to decrease (7.7 mmol/L for reference drink and 5.5 mmol/L for test drink) and both returned to baseline at the last blood glucose measurement (5.6 mmol/L for reference drink and 4.7 mmol/L for test drink). This response was the same as those reported in studies testing blood glucose response to fruits such as watermelon, papaya and durian [16]. The decrease in glucose absorption 2-h post prandial is due to counter regulatory by hormones such as insulin and glucagon [6, 17]. The urine dipstick results for all 10 participants were negative at all sampling time points in all of the tests.

The GI for the test drink (calamansi) was estimated as 37, showing that it has a low potential for raising blood glucose levels which can be inferred to cause a similar albeit slightly higher response in a diabetic individual [18, 19]. Food with low GI may slow the rate of gastric emptying as it is associated with prolonged small intestine transit time that reduces postprandial glucose absorption [20, 21]. The acidity contributed by the citric acid and ascorbic acid content in the calamansi drink, could also be a factor as acidity caused delayed gastric emptying [22]. The low GI could also be due to the content of the calamansi drink tested as calamansi fruit skin is rich in flavonoids [23] such as hesperidin and naringin that have been suggested to have hypoglycaemic properties in vitro [24] and in a diabetic rat model [8]. The fruit skin of calamansi also contains pectin, a natural fibre that can decrease the rise in blood glucose levels following a meal by shortening glucose contact time with the absorbing surface [25].

## Limitations

- The sample population. Future studies should also include pre-diabetic individuals and diabetic patients to enable comparison with demographic data.
- The test drink. Although freshly prepared test drink would have been ideal, this was not feasible due to logistical constraints and this led to the choice of a commercially available drink.
- The gender of the study participants. Future research should include both genders so that it is more representative of the general population.

## Data Availability

This could be made available from the corresponding author upon reasonable request.

## Abbreviations

BMI: Body mass index
FAO: Food and Agriculture Organization
FFA: Free fatty acids
GI: Glycaemic index
HIV: Human immunodeficiency virus
IAUC: Incremental area under the curve
SE: Standard error
WHO: World Health Organisation
